# Optic Disc and Macular Vessel Density Measured by Optical Coherence Tomography Angiography in Open-Angle and Angle-Closure Glaucoma

**DOI:** 10.1038/s41598-020-62633-4

**Published:** 2020-03-27

**Authors:** Tzu-Yu Hou, Tung-Mei Kuang, Yu-Chieh Ko, Yu-Fan Chang, Catherine Jui-Ling Liu, Mei-Ju Chen

**Affiliations:** 1 Department of Ophthalmology, Taipei Veterans General Hospital, Taipei, Taiwan; 2 Faculty of Medicine, National Yang-Ming University School of Medicine, Taipei, Taiwan

**Keywords:** Pathogenesis, Circulation

## Abstract

There is distinct pathogenesis between primary open-angle glaucoma (POAG) and primary angle-closure glaucoma (PACG). Although elevated intraocular pressure (IOP) is the major risk factor for glaucoma, non-IOP risk factors such as vascular abnormalities and lower systolic/diastolic perfusion pressure may play a role in the pathogenic process. This study aimed to compare the vessel density (VD) in the optic disc and macula using optical coherence tomography angiography (OCTA) between POAG and PACG eyes. Thirty-two POAG eyes, 30 PACG eyes, and 39 control eyes were included. All the optic disc VD parameters except the inside disc VD were significantly lower in glaucomatous eyes than in control eyes. Compared with PACG eyes, only the inferior temporal peripapillary VD was significantly lower in POAG eyes. The parafoveal VD was significantly lower in each quadrant in glaucomatous eyes than in control eyes. The central macular and parafoveal VD did not differ between POAG and PACG eyes. In conclusion, the inferior temporal peripapillary VD was significantly reduced in POAG eyes compared with PACG eyes, while PACG eyes showed a more evenly distributed reduction in the peripapillary VD. The distinct patterns of VD change may be associated with the different pathogenesis between POAG and PACG.

## Introduction

Glaucoma is an optic neuropathy characterised by progressive loss of retinal ganglion cells and their axons accompanied by corresponding visual field (VF) defects. Primary glaucoma is classified according to the anatomy of the anterior chamber angle into primary open-angle glaucoma (POAG) and primary angle-closure glaucoma (PACG). Elevated intraocular pressure (IOP) is the major risk factor for glaucoma. In PACG, elevated IOP secondary to angle closure is considered the primary mechanism. On the other hand, other non-IOP risk factors such as vascular abnormalities and lower systolic/diastolic perfusion pressure have been proposed in POAG^1–4^. The characteristics of the optic disc are also different between POAG and PACG eyes. There may be enlarged cupping and/or optic disc rim notching in POAG eyes, whereas pallor of the optic disc either from an acute attack of angle closure or in the chronic clinical course may be observed in PACG eyes^5,6^. All these findings indicate the distinct pathogenesis between POAG and PACG and reflect the feature of microvascular damage.

Optical coherence tomography angiography (OCTA) is a reliable technique to perform *in vivo* imaging of the optic nerve head (ONH) and retinal microcirculation^7–11^. Previous studies have reported reduced vessel density (VD) in the ONH, peripapillary area, and macula in glaucomatous eyes^12–17^. Most of the studies investigated POAG eyes and had limited analyses for the microcirculation in PACG eyes^18–21^. The diagnostic ability of VD as well as the relationship of peripapillary VD with VF and/or retinal nerve fibre layer (RNFL) thickness in POAG and PACG have been reported^18–28^. To date, no reports have compared the pattern of regional VD change in the optic disc or macula between PACG and POAG. Thus, we aimed to compare the optic disc and macular VD in each sector as well as the pattern of VD change between POAG and PACG. In addition, we tried to illustrate the different microvascular contribution to the pathogenesis of POAG and PACG.

## Results

This study included 32 POAG eyes, 30 PACG eyes, and 39 control eyes. Eleven eyes (36.7%) in the PACG group had a history of an acute attack. In the POAG group, 15 eyes (46.9%) were normal tension glaucoma (NTG) with an untreated baseline IOP <21 mmHg, and the other 17 eyes (53.1%) were high tension glaucoma (HTG) with an untreated baseline IOP ≥21 mmHg. Among the 32 POAG eyes, 4 were not on any anti-glaucoma medications, 14 were on topical beta blockers, 7 were on alpha agonists, 6 were on carbonic anhydrase inhibitors, and 18 were on prostaglandin analogues (either as a monotherapy or as an individual component in a combination therapy). Among the 30 PACG eyes, 9 were not on any anti-glaucoma medications, 13 were on topical beta blockers, 10 were on alpha agonists, 2 were on carbonic anhydrase inhibitors, and 11 were on prostaglandin analogues (either as a monotherapy or as an individual component in a combination therapy). The demographics and clinical characteristics of the subjects were shown in Table 1. There was no significant difference in age, best-corrected visual acuity (BCVA), IOP, central corneal thickness (CCT), systolic blood pressure (SBP), or the proportion of subjects having systemic diseases (i.e., hypertension and cardiovascular disease) when comparing each pair from the three groups. Female subjects were predominant in the PACG group. Diastolic blood pressure (DBP) was not significantly different when comparing either the POAG and control groups (p = 0.966) or the POAG and PACG groups (p = 0.066) but significantly lower in the PACG group compared with the control group (p = 0.041). The spherical equivalence (SE) was not significantly different between the PACG and control groups (p = 0.977) or the POAG and PACG groups (p = 0.093). However, the eyes were more myopic in the POAG group than in the control group (p = 0.016). The average number of anti-glaucoma medications and the VF parameters, including mean deviation (MD), pattern standard deviation (PSD), and VF index, did not differ between the POAG and PACG groups.

**Table 1:** Demographics and clinical characteristics of included subjects.

	Control (n = 39)	POAG (n = 32)	PACG (n = 30)	p*	p**	p***
Age (years)	69.08 ± 5.03	67.16 ± 6.04	70.47 ± 5.66	0.329	0.542	0.075
Sex (male/female)	14/25	19/13	3/27	0.059	0.023	0.000
Hypertension, % (n)	41.03%	62.50%	36.67%	0.096	0.806	0.074
Cardiovascular disease, % (n)	12.82%	29.03%	16.67%	0.133	0.737	0.363
SBP (mmHg)	138.29 ± 19.54	142.65 ± 20.11	132.65 ± 21.45	0.758	0.599	0.298
DBP (mmHg)	81.64 ± 15.18	82.88 ± 16.63	70.91 ± 15.29	0.966	0.041	0.066
BCVA	0.86 ± 0.13	0.82 ± 0.20	0.77 ± 0.22	0.537	0.105	0.631
SE (D)	0.49 ± 1.67	−0.96 ± 2.30	0.38 ± 2.41	0.016	0.977	0.093
IOP (mmHg)	15.77 ± 3.50	16.23 ± 3.07	16.03 ± 3.61	0.831	0.950	0.973
CCT (μm)	541.48 ± 32.96	555.32 ± 26.74	551.21 ± 35.62	0.261	0.586	0.897
Glaucoma eyedrops (n)	0.23 ± 0.48	1.41 ± 0.98	1.20 ± 0.96	0.000	0.000	0.682
Visual field index (%)	96.33 ± 7.87	88.06 ± 9.79	91.43 ± 6.43	0.001	0.016	0.249
Visual field MD (dB)	−0.16 ± 3.33	−4.31 ± 3.46	−4.46 ± 3.37	0.000	0.000	0.982
Visual field PSD (dB)	2.21 ± 1.96	5.67 ± 3.52	4.00 ± 2.25	0.000	0.003	0.075

Values are presented as mean ± standard deviation unless otherwise indicated. *Comparison between the control and POAG groups. **Comparison between the control and PACG groups. ***Comparison between the POAG and PACG groups. BCVA, best-corrected visual acuity; CCT, central corneal thickness; dB, decibel; DBP, diastolic blood pressure; IOP, intraocular pressure; MD, mean deviation; PACG, primary angle-closure glaucoma; POAG, primary open-angle glaucoma; PSD, pattern standard deviation; SBP, systolic blood pressure; SE, spherical equivalence.

The average and sector circumpapillary retinal nerve fibre layer (cpRNFL) thickness values were significantly lower in glaucomatous eyes than in control eyes except for the 3, 4, and 9 clock-hour sectors (Table 2). The 6 and 7 clock-hour sector cpRNFL thickness values were significantly thinner in the POAG group than in the PACG group (p = 0.020 and p < 0.001, respectively). Otherwise, the cpRNFL thickness in the other sectors did not show significant difference between the POAG and PACG groups. The macular ganglion cell inner plexiform layer (GCIPL) thickness values were thinner in all the sectors in the POAG group as well as in the inferior and inferior temporal sectors in the PACG group compared with the control group (Table 2). Compared with the PACG group, the macular GCIPL thickness values were thinner in the inferior nasal, inferior, and inferior temporal sectors in the POAG group (p = 0.035, p = 0.002, and p < 0.001, respectively).

**Table 2:** Comparison of the circumpapillary retinal nerve fibre layer and macular ganglion cell inner plexiform layer thickness measurements among three groups.

	Control (n = 39)	POAG (n = 32)	PACG (n = 30)	p*	p**	p***
Average cpRNFL thickness (μm)	94.87 ± 8.76	71.59 ± 11.73	75.87 ± 15.55	0.000	0.000	0.449
12-sector cpRNFL thickness (μm)						
12 clock-hour	125.79 ± 37.41	93.75 ± 27.19	97.67 ± 26.67	0.000	0.002	0.835
1 clock-hour	108.97 ± 27.17	84.72 ± 21.16	83.73 ± 24.78	0.000	0.000	0.985
2 clock-hour	79.79 ± 12.99	69.88 ± 11.35	69.93 ± 15.04	0.003	0.016	1.000
3 clock-hour	60.64 ± 9.80	59.88 ± 12.41	58.57 ± 14.33	0.957	0.776	0.922
4 clock-hour	61.92 ± 9.19	60.47 ± 13.71	59.17 ± 14.54	0.865	0.638	0.930
5 clock-hour	95.08 ± 21.74	70.09 ± 21.09	75.67 ± 22.55	0.000	0.002	0.578
6 clock-hour	130.13 ± 24.02	74.66 ± 22.64	91.43 ± 24.70	0.000	0.000	0.020
7 clock-hour	140.69 ± 20.61	72.63 ± 26.98	103.83 ± 24.17	0.000	0.000	0.000
8 clock-hour	71.69 ± 14.13	57.75 ± 18.55	61.07 ± 11.24	0.003	0.003	0.669
9 clock-hour	57.95 ± 9.25	52.09 ± 14.72	53.13 ± 10.64	0.134	0.129	0.945
10 clock-hour	83.54 ± 17.89	66.81 ± 26.27	71.27 ± 17.52	0.009	0.016	0.711
11 clock-hour	120.82 ± 21.52	94.63 ± 32.76	97.8 ± 29.76	0.001	0.002	0.916
Macular GCIPL thickness (μm)						
Average	79.72 ± 13.42	66.66 ± 10.12	73.70 ± 8.69	0.000	0.070	0.013
Superior	80.03 ± 13.48	69.59 ± 13.07	75.47 ± 11.49	0.004	0.291	0.153
Superior nasal	83.05 ± 14.55	71.59 ± 13.24	76.73 ± 10.37	0.003	0.096	0.211
Inferior nasal	80.03 ± 14.01	67.38 ± 11.35	74.27 ± 9.86	0.000	0.120	0.035
Inferior	78.61 ± 7.98	61.16 ± 10.32	70.00 ± 9.48	0.000	0.001	0.002
Inferior temporal	79.21 ± 13.26	61.53 ± 8.97	71.70 ± 10.77	0.000	0.031	0.000
Superior temporal	79.26 ± 11.94	68.44 ± 10.76	73.97 ± 10.84	0.000	0.141	0.117

Values are presented as mean ± standard deviation unless otherwise indicated. *Comparison between the control and POAG groups (Games-Howell post-hoc test). **Comparison between the control and PACG groups (Games-Howell post-hoc test). ***Comparison between the POAG and PACG groups (Games-Howell post-hoc test). cpRNFL, circumpapillary retinal nerve fibre layer; GCIPL, ganglion cell inner plexiform layer; PACG, primary angle-closure glaucoma; POAG, primary open-angle glaucoma.

**Figure 1:**
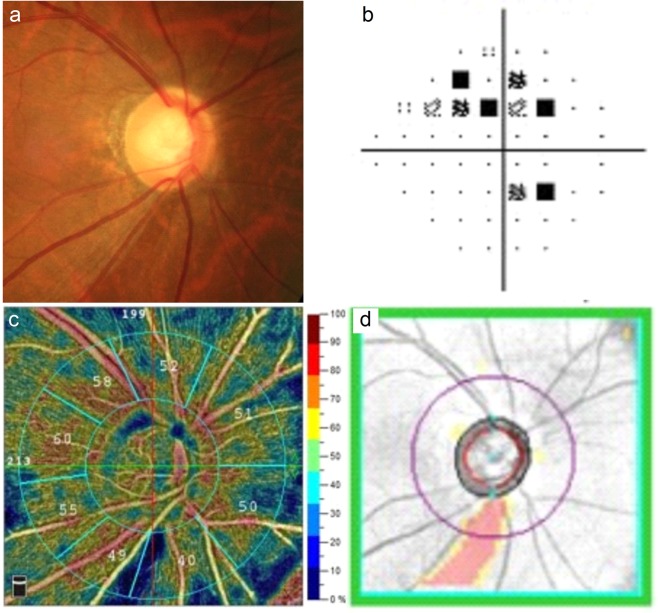
Images from the right eye of an individual with primary open-angle glaucoma. (**a**) The optic disc photography shows characteristic vertical elongation of the optic cup accompanied by a loss of inferior temporal circumpapillary retinal nerve fibre layer (cpRNFL). (**b**) The visual field test shows a superior arcuate defect with a mean deviation of −2.46 decibels. (**c**) The optical coherence tomography angiography (RTVue XR with AngioVue; version 2018.0.0.14; URL: http://www.optovue.com) shows reduced vessel densities in the inferior temporal sector of radial peripapillary capillary image. (**d**) The Cirrus optical coherence tomography shows inferior temporal cpRNFL thinning corresponding to that presenting in the optic disc photography.

**Figure 2:**
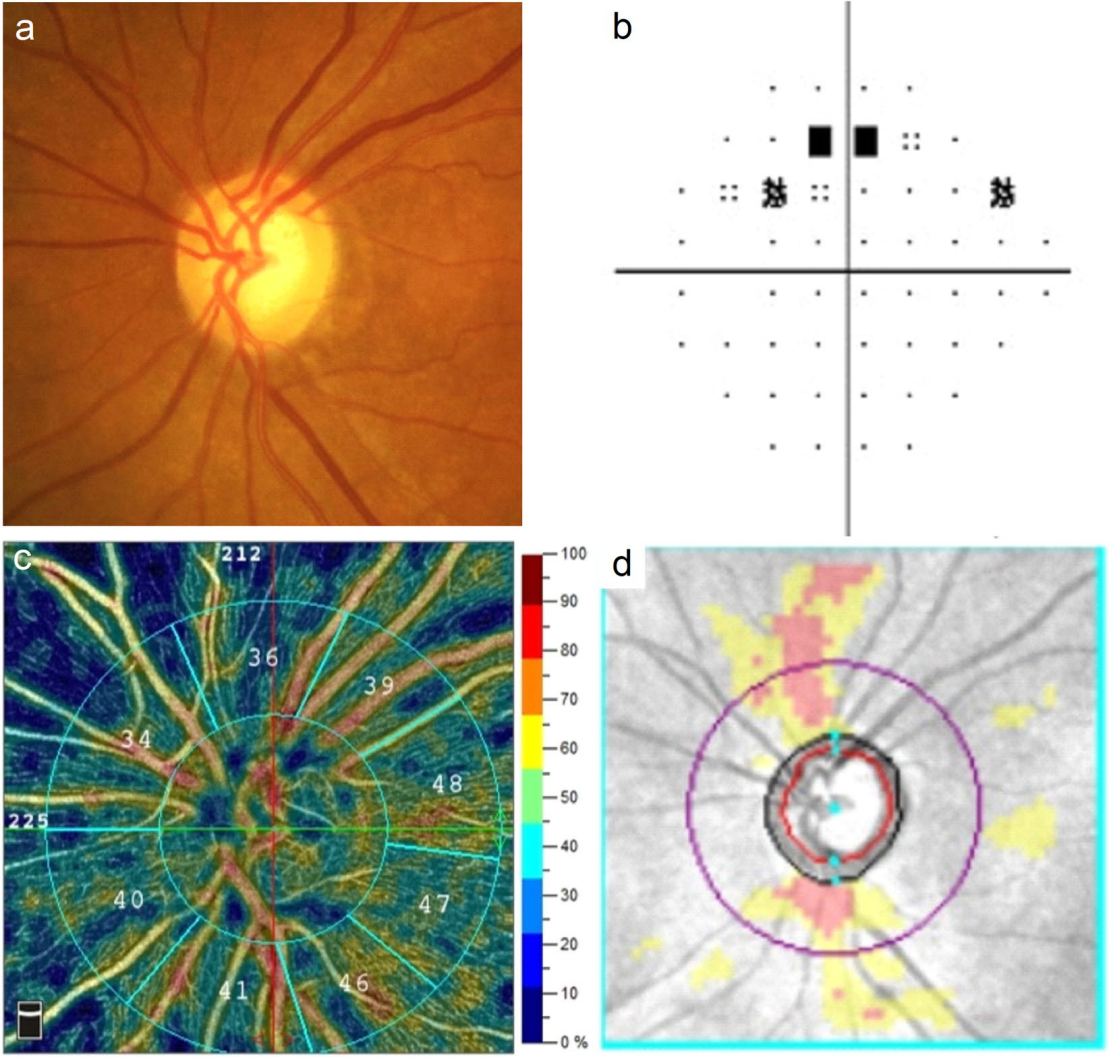
Images from the left eye of an individual with primary angle-closure glaucoma. (**a**) The optic disc photography shows characteristic glaucomatous changes. (**b**) The visual field test shows a superior arcuate defect with a mean deviation of −3.33 decibels. (**c**) The optical coherence tomography angiography (RTVue XR with AngioVue; version 2018.0.0.14; URL: http://www.optovue.com) shows a generalised reduction of peripapillary vessel densities. (**d**) The Cirrus optical coherence tomography shows retinal nerve fibre layer thinning corresponding to visual field defects.

For the optic disc VD, almost all the parameters (i.e., whole image and all sector peripapillary VD) were significantly lower in glaucomatous eyes than in control eyes, except for the inside disc VD (Table 3). Compared with the PACG group, the inferior temporal peripapillary VD was significantly lower in the POAG group (p < 0.001) (Table 3; Figs. 1 and 2). The remaining VD parameters in the optic disc area did not differ between the POAG and PACG groups.

**Table 3:** Comparison of the optic disc vessel density among three groups.

	Control (n = 39)	POAG (n = 32)	PACG (n = 30)	p*	p**	p***
Average capillary VD (%)						
Whole image	47.90 ± 1.98	39.96 ± 5.18	41.50 ± 4.55	0.000	0.000	0.429
Inside disc	48.03 ± 5.08	45.58 ± 7.17	45.68 ± 5.34	0.243	0.164	0.997
Peripapillary	50.84 ± 2.03	40.84 ± 6.22	43.70 ± 5.85	0.000	0.000	0.158
8-sector peripapillary VD (%)						
Superior	50.44 ± 4.21	41.25 ± 9.75	41.53 ± 9.72	0.000	0.000	0.993
Superior nasal	47.10 ± 3.64	39.66 ± 7.08	38.63 ± 8.29	0.000	0.000	0.861
Inferior nasal	45.33 ± 4.14	39.66 ± 7.44	38.97 ± 7.46	0.001	0.000	0.930
Inferior	49.23 ± 4.45	35.47 ± 10.72	40.39 ± 8.78	0.000	0.000	0.133
Inferior temporal	57.90 ± 3.56	33.81 ± 11.47	49.59 ± 7.92	0.000	0.000	0.000
Temporal lower	50.95 ± 3.49	44.84 ± 8.01	46.47 ± 6.94	0.001	0.007	0.674
Temporal upper	55.41 ± 2.72	48.55 ± 7.95	51.50 ± 6.48	0.000	0.010	0.257
Superior temporal	53.41 ± 8.02	43.00 ± 10.32	45.57 ± 9.18	0.000	0.001	0.557

Values are presented as mean ± standard deviation unless otherwise indicated. *Comparison between the control and POAG groups (Games-Howell post-hoc test). **Comparison between the control and PACG groups (Games-Howell post-hoc test). ***Comparison between the POAG and PACG groups (Games-Howell post-hoc test). VD, vessel density; PACG, primary angle-closure glaucoma; POAG, primary open-angle glaucoma.

For the macular area, the parafoveal VD was significantly lower in each quadrant in glaucomatous eyes than in control eyes, whereas the central macular VD did not differ between glaucomatous and control eyes (Table 4). When comparing the POAG and PACG groups, the VD in all macular regions did not show significant difference.

**Table 4:** Comparison of the macular vessel density among three groups.

	Control (n = 39)	POAG (n = 32)	PACG (n = 30)	p*	p**	p***
Central VD (%)	14.31 ± 5.40	14.16 ± 4.18	12.70 ± 5.00	0.990	0.413	0.435
Quadrant parafoveal VD (%)						
Superior	47.23 ± 4.25	41.88 ± 6.28	43.07 ± 5.28	0.000	0.002	0.698
Nasal	46.05 ± 5.14	41.03 ± 5.41	42.00 ± 4.43	0.001	0.002	0.721
Inferior	47.92 ± 4.18	40.25 ± 6.70	43.63 ± 4.80	0.000	0.001	0.065
Temporal	46.13 ± 3.55	39.06 ± 5.57	42.00 ± 4.76	0.000	0.001	0.073

Values are presented as mean ± standard deviation unless otherwise indicated. *Comparison between the control and POAG groups (Games-Howell post-hoc test). **Comparison between the control and PACG groups (Games-Howell post-hoc test). ***Comparison between the POAG and PACG groups (Games-Howell post-hoc test). VD, vessel density; PACG, primary angle-closure glaucoma; POAG, primary open-angle glaucoma.

## Discussion

Our study showed that the whole optic disc, peripapillary, and parafoveal VD were significantly reduced in POAG and PACG eyes compared with control eyes, indicating compromised retinal vascular perfusion in glaucomatous eyes. This finding was consistent with previous reports that the retinal microcirculation was impaired in peripapillary and parafoveal regions in glaucomatous eyes^12–22^. In addition, the diagnostic ability of retinal VD has been proved in both POAG and PACG^20–25^. However, no studies have directly compared the difference in the optic disc or macular VD between POAG and PACG.

In the present study, the inferior temporal peripapillary VD was significantly lower in POAG eyes than in PACG eyes despite similar BCVA, IOP, CCT, and VF parameters in these two groups. However, PACG eyes showed a more evenly distributed reduction of VD. Our findings were in accordance with earlier reports that the inferior temporal VD reduced most and had the highest diagnostic ability among the peripapillary sectors in POAG eyes^24,26,29^. The difference in the peripapillary VD between POAG and PACG groups corresponded to the pattern of cpRNFL loss (Tables 2 and 3). Previous studies have found the inferior temporal peripapillary VD to have the strongest association with the corresponding cpRNFL thickness and visual sensitivity loss in POAG eyes^27,30^. Holló^26^ even suggested that measuring the inferior temporal peripapillary angioflow density could identify glaucomatous damage earlier than measuring the corresponding RNFL thickness. Thus, by analysing the inferior temporal peripapillary VD, POAG eyes may be distinguished not only from control eyes but also from PACG eyes.

The distinct patterns of peripapillary VD between POAG and PACG eyes may be associated with the different pathophysiologies of the two disease entities. The optic disc is characterised by localised rim notching in POAG eyes, while it appeared to be pallor after acute angle closure or in chronic PACG^5,6^. The earliest glaucomatous sign in POAG is localised inferior or inferotemporal cpRNFL thinning, which correlates with the VF pattern of localised defects^31–33^. Conversely, the VF damage tends to be diffuse in PACG^34,35^. The preference for the inferior temporal sector of glaucomatous optic neuropathy in POAG has been related to the larger single pores of the inferior temporal lamina cribrosa and the least supporting connective tissue in this region^36,37^.

On the other hand, PACG is characterised by trabecular outflow obstruction accompanied by intermittent IOP spikes and a wide range of diurnal IOP fluctuations^34,38^. In the present study, 11 eyes (36.7%) in the PACG group had a history of an acute attack. Elevated IOP may subsequently result in ischemia of the optic disc^22^. Zhang *et al*.^18^ reported significantly reduced peripapillary retinal VD in eyes having experienced acute angle closure compared with the fellow eyes. They suggested that acute IOP elevation had a detrimental effect on the VD. Lee *et al*.^39^ found more diffuse cpRFNL damage in eyes with non-arteritic anterior ischemic optic neuropathy (NAION) compared with eyes with POAG. Elevated IOP may cause an ischemic insult in PACG similar to NAION, leading to generalised reduction of the peripapillary VD^39,40^. This effect, however, is less determined in the pathogenesis of POAG. Jo *et al*.^28^ reported that the association between IOP elevation and peripapillary VD reduction was presented only in PACG eyes but not in POAG eyes. Therefore, the pattern of peripapillary VD change may differ between POAG and PACG.

There was no significant difference in the inside disc VD between each pair from the three groups (Table 3). Rao *et al*.^24^ reported poor diagnostic abilities of the inside disc VD compared with the peripapillary VD. Thus, the peripapillery VD may by a better indicator for the evaluation of vascular change in POAG and PACG.

The central macular and parafoveal VD did not differ between POAG and PACG eyes (Table 4). The parafoveal and peripapillary microvasculature may differ in the blood supply, size of the vessel, peak capillary density profile, and response to elevated IOP. The peripapillary region is supplied by the central retinal artery and ciliary arteries, while the macula is supplied by the central retinal artery alone. The peripapillary vasculature includes four main arteries and veins, while the parafoveal region comprises capillary network and small vessels. Using a projection-resolved OCTA algorithm, the peak capillary density in the superficial vascular plexus was shown to be higher in the peripapillary region than in the parafoveal region^41^. Hayreh *et al*.^42^ reported that the choroidal circulation of the optic disc was most susceptible to high IOP. Highly elevated IOP may cause either virtual obliteration of the optic disc and peripapillary choroid or simply slowing of the retinal circulation^42^. In PACG eyes, the VD reduces greater in the peripapillary region than in the parafoveal region^20,22^. Besides, the diagnostic ability of the macular VD has been found to be lower than the peripapillary VD, the cpRNFL thickness, and the macular GCIPL thickness^23–25,43^. These aforementioned findings indicate that the glaucomatous vascular change is less sensitive in the macula than in the peripapillary region. Therefore, the difference in the macular VD between POAG and PACG eyes may not be detected.

In the present study, the POAG group included NTG eyes (46.9%) and HTG eyes (53.1%). Xu *et al*.^29^ reported that NTG eyes had significantly lower retinal VD than HTG eyes in all peripapillary sectors except the inferior temporal sector. Scripsema *et al*.^44^ demonstrated lower peripapillary VD in HTG eyes than in NTG eyes, while Bojikian *et al*.^45^ reported no difference in the optic disc VD between HTG and NTG eyes for the same level of VF loss. We did not perform the subgroup analysis because of the small sample size. Due to contrasting results among previous studies, further research is warranted to investigate the difference in microvascular dysfunction between NTG and HTG eyes.

More myopia was presented in POAG eyes than in control eyes (Table 1). Triolo *et al*.^25^ reported that no OCTA parameters were correlated with SE. Suwan *et al*.^46^ reported lower peripapillary perfused capillary densities in either myopic eyes compared with control eyes or myopic POAG eyes compared with non- myopic POAG eyes. However, the mean SE in their study was more than −5.0 dioptres (D) in myopic eyes. In our study, the mean SE was −0.96 D in POAG eyes. Thus, the influence on retinal microcirculation might be negligible.

Our study had several limitations. First, the sample size was relatively small, which was partly due to difficulties in dilating the pupil to ensure qualified OCTA images in PACG eyes. Second, the subjects were all Chinese. Therefore, our results may not be generalisable to other races. Third, this cross-sectional study could not determine if the reduced VD resulted primarily from vascular events or from glaucomatous structural changes. Fourth, our study used 3 × 3 mm^2^ imaging of the macula. This area may be too small to adequately sample retinal vascular changes in glaucoma. Fifth, reduced macular VD and increased foveal avascular zone in patients with hypertension have been reported^47,48^. However, the optic disc VD was shown to reduce only in patients with first diagnosis of systemic hypertension but not in patients already treated for systemic hypertension^49^. Wang *et al*.^48^ also reported decreased retinal and choroidal VD in the macular region in patients with coronary heart disease. In our study, only subjects with treated hypertension and mild cardiovascular diseases rather than coronary artery disease were enrolled. The number of subjects with hypertension and the number of subjects with cardiovascular diseases were equally distributed in the 3 study groups. Therefore, the effect on the macular VD might be minimal. Finally, although an association between topical beta blockers and macular VD has been reported, the effect of topical anti-glaucoma medications on retinal or optic disc blood circulation is inconclusive^17,50^. Besides, none of the other topical medications (i.e., alpha agonists, carbonic anhydrase inhibitors, and prostaglandin analogues) were shown to have significant influence on the retinal or optic disc VD^17^. In this study, the number of anti-glaucoma medications was similar between the POAG and PACG groups. Further research is necessary to evaluate the effect of anti-glaucoma medications on VD obtained from OCTA.

In conclusion, our study demonstrated significantly lower VD in the whole image of optic disc, peripapillary sectors, and parafoveal quadrants in glaucomatous eyes than in normal eyes. POAG eyes showed a significant reduction in the inferior temporal peripapillary VD compared with PACG eyes, while PACG eyes showed a more evenly distributed loss of the peripapillary VD. The regional difference in VD between POAG and PACG eyes may enhance our knowledge in the pathogenesis of glaucoma.

## Methods

### Subjects

Patients with POAG or PACG who visited the outpatient clinic of Taipei Veterans General Hospital between May 2018 and August 2018 were recruited for this study. Also, age-matched control subjects were enrolled by recruiting healthy volunteers from the same hospital^51^. The study protocol was approved by the Institutional Review Board of Taipei Veterans General Hospital and was designed in accordance with the Declaration of Helsinki. Written informed consent was obtained from all subjects.

Glaucomatous eyes were defined as eyes with focal or diffuse RNFL defects corresponding to glaucomatous optic disc changes and VF defects^51^. Glaucomatous optic disc changes were defined as a >0.7 vertical cup-to-disc ratio (C/D); a >0.2 asymmetric C/D between the glaucomatous and normal eyes; and neuroretinal rim thinning, notching, or excavation on optic disc photography^51^. Focal or diffuse RNFL defects were identified on the red-free fundus image. All images, including the ONH photograph and RNFL thickness scan, were evaluated by glaucoma specialists who were blinded to the information from the subjects’ clinical evaluation. A glaucomatous VF was defined as three contiguous, non-edge points within the same hemifield with a p-value <0.05 for the PSD as well as at least one point with a p-value <0.01, and/or outside normal limits in the glaucoma hemifield test^52^. A reliable VF test was defined as having a fixation loss rate of <20%, a false positive rate of <33%, and a false negative rate of <33%.

All participants underwent a comprehensive ophthalmic examination, including BCVA, automated refraction and keratometry, Goldmann applanation tonometry, slit-lamp examination, gonioscopy, dilated fundus examination, red-free fundus photography, 24–2 SITA standard algorithm automated VF examination using the Humphrey Visual Field Analyser (model 720i, Zeiss Humphrey Systems, Dublin, California, USA), and CCT determined by the DGH 55 Pachmate (DGH Technology, Exton, Pennsylvania, USA). The inclusion criteria for all participants were as follows: age ≥ 20 years; BCVA ≥ 20/40; and refractive error within ±6 dioptres (D) sphere and ±3 D cylinder. Control subjects had a normal anterior segment on the slit-lamp examination without glaucomatous ONH changes or VF defects. POAG eyes had open anterior chamber angles, while PACG eyes had occludable anterior chamber angles in three or more quadrants. An occludable anterior chamber angle was defined as one in which the trabecular meshwork was seen in less than 90 degrees of the angle circumference by gonioscopy. Only eyes with early to moderate glaucomatous damage and the VF MD ≥ −12.0 decibels (dB), in accordance with Hodapp’s classification, were included in the glaucoma groups^53^. Eyes with retinal or neurologic diseases, media opacities, ocular inflammation, ocular surgery within 3 months prior to the examination date, prior refractive surgery, or concurrent diseases that may interfere with OCTA imaging or lead to VF defects were excluded^22^. Eyes in subjects with diabetes mellitus, first diagnosis of systemic hypertension, or coronary artery disease were also excluded.

### OCT examination

The Cirrus HD-OCT (Carl Zeiss Meditec, Dublin, California, USA) was performed following pupillary dilation. The Cirrus HD-OCT Optic Disc Cube 200 × 200 protocol was used to measure the average and 12-sector cpRNFL thickness. The circumpapillary sector was named according to 12 clock hours in a clockwise direction in the right eye and in a counterclockwise direction in the left eye with the superior sector designated as 12 o’clock^21^. The Macular Cube 200 × 200 protocol was used to calculate the average and 6-sector parafoveal GCIPL thickness. Images with signal strength <7, motion artifacts, poor centration, segmentation errors, artifacts from ocular pathologies, or missing data in the peripapillary region were excluded.

### OCTA examination

The OCTA was performed using RTVue-XR spectral domain OCT (AngioVue, Optovue Inc., Fremont, California, USA; version 2018.0.0.14; URL: http://www.optovue.com). The optic disc scan covered an area of 4.5 × 4.5 mm^2^ centred on the optic disc, while the macular scan covered an area of 3 × 3 mm^2^ centred on the fovea. The VD was defined as the proportion of the total area occupied by blood vessels. A blood vessel was defined as pixels having decorrelation values in the noise region exceeding the threshold value by two standard deviations above the average decorrelation value^12^. In the optic disc scan, the software automatically calculated the whole image VD (covering an area of 4.5 × 4.5 mm^2^), average VD within the ONH (inside disc VD), and peripapillary VD (measured in a 750 um-wide annulus extending outward from the optic disc boundary). In the macular scan, the parafoveal VD was measured in an annulus centred on the fovea with an outer diameter of 3 mm and an inner diameter of 1 mm. The peripapillary VD was analysed from the radial peripapillary capillary segment, extending from the internal limiting membrane (ILM) to the posterior boundary of the RNFL. The macular VD was analysed from the superior vascular plexus between the ILM and the inner plexiform layer. The peripapillary region was divided into eight sectors of 45 degrees each (i.e., superior, superior nasal, inferior nasal, inferior, inferior temporal, temporal lower, temporal upper, and superior temporal sectors)^20^. The macular area included one central macular region and four parafoveal quadrants of 90 degrees each (i.e., superior, temporal, inferior, and nasal quadrants). Images with poor quality (defined as having a signal strength index <5), segmentation errors, or any residual motion artifacts were excluded. The time interval between OCTA and other ophthalmic examinations (e.g., VF) was less than 3 months. All OCT and OCTA examinations were measured by the same experienced technician.

### Statistical analysis

For each subject, only one eye was analysed. If both eyes were eligible, one eye was randomly chosen. Statistical analyses were performed using SPSS version 19.0 (SPSS, Inc., Chicago, IL, USA). The data were presented as the mean ± standard deviation. For continuous variables, the normality of data distribution was verified using the Shapiro-Wilk test. The analysis of variance (ANOVA) with Games-Howell post-hoc test was used to analyse the difference in demographics, OCT and OCTA parameters. For categorical variables, the Chi-square test was used to compare the study subjects. A p-value <0.05 was considered statistically significant.

## Data Availability

Datasets from the current study are not publicly available due to compliance to privacy. Summary statistics are available from the corresponding author on reasonable request.
